# Entry of cannabidiol into the fetal, postnatal and adult rat brain

**DOI:** 10.1007/s00441-024-03867-w

**Published:** 2024-02-17

**Authors:** Georgia Fitzpatrick, Yifan Huang, Fiona Qiu, Mark D. Habgood, Robert L. Medcalf, Heidi Ho, Katarzyna M. Dziegielewska, Norman R. Saunders

**Affiliations:** Department of Neuroscience, Monash University, Melbourne, VIC 3004 Australia

**Keywords:** Blood brain and placental barriers, Cerebrospinal fluid, Drug transfer, Cannabidiol receptors, Plasma protein binding

## Abstract

Cannabidiol is a major component of cannabis but without known psychoactive properties. A wide range of properties have been attributed to it, such as anti-inflammatory, analgesic, anti-cancer, anti-seizure and anxiolytic. However, being a fairly new compound in its purified form, little is known about cannabidiol brain entry, especially during development. Sprague Dawley rats at four developmental ages: embryonic day E19, postnatal day P4 and P12 and non-pregnant adult females were administered intraperitoneal cannabidiol at 10 mg/kg with [^3^H] labelled cannabidiol. To investigate the extent of placental transfer, the drug was injected intravenously into E19 pregnant dams. Levels of [^3^H]-cannabidiol in blood plasma, cerebrospinal fluid and brain were estimated by liquid scintillation counting. Plasma protein binding of cannabidiol was identified by polyacrylamide gel electrophoresis and its bound and unbound fractions measured by ultrafiltration. Using available RNA-sequencing datasets of E19 rat brain, choroid plexus and placenta, as well as P5 and adult brain and choroid plexus, expression of 13 main cannabidiol receptors was analysed. Results showed that cannabidiol rapidly entered both the developing and adult brains. Entry into CSF was more limited. Its transfer across the placenta was substantially restricted as only about 50% of maternal blood plasma cannabidiol concentration was detected in fetal plasma. Albumin was the main, but not exclusive, cannabidiol binding protein at all ages. Several transcripts for cannabidiol receptors were expressed in age- and tissue-specific manner indicating that cannabidiol may have different functional effects in the fetal compared to adult brain.

**Supplementary Information:**

The online version contains supplementary material available at 10.1007/s00441-024-03867-w.

## Introduction

Many therapeutic (Briggs et al. 2021) as well as recreational (Goodwin et al. 2021) and illegal drugs of addiction (Smith et al. 2015; Jantzie et al*.* 2020) are taken by pregnant women in spite of a considerable lack of information on the extent to which most of them can cross the placenta, enter the fetus and the fetal brain (Saunders and Dziegielewska 2020; Couzin-Frankel 2022). This is because it has been considered unethical to conduct clinical trials in expectant mothers or young children (Sinclair et al. 2016) and animal studies have been limited until recently (e.g. Koehn et al. 2019a, 2021; Saunders and Dziegielewska 2020; Toll et al. 2021; Huang et al. 2023). For clinically significant conditions such as epilepsy, depression, anxiety and psychosis in the mother, it may be important that women continue their medication in spite of uncertainty about potential risks to the baby. In the case of recreational and illegal drugs, the situation is more complicated because women taking these drugs may be reluctant to disclose their use to medical staff. A number of epidemiological studies have reported associations between the drug taken by the mother and unfavourable behavioural outcomes in the offspring (e.g. acetaminophen/paracetamol, Liew et al. 2014, Baker et al. 2022, Nilsen et al*.* 2023; anti-seizure medications, Wood et al. 2015, Knight et al. 2021).

Cannabis is a common psychoactive drug used historically for both recreational and medicinal purposes (Abel 1980; Crocq 2020). It is often used medicinally for its analgesic, anxiolytic and anti-seizure properties (Berger et al. 2022; Henderson et al. 2021; Amin and Ali 2019). Cannabis contains two main active cannabinoids, tetrahydrocannabinol (THC), the psychoactive constituent and cannabidiol (CBD) which has no psychoactive effects (Martin 2020; Ribeiro Do Valle 1969). Use of cannabidiol has been suggested to cause a broad range of claimed outcomes, from anti-inflammation and analgesia (Atalay et al. 2019; Costa et al. 2004; Nagarkatti et al. 2009), to anti-cancer (Massi et al. 2004; O’Brien 2022; Ramer et al. 2013; Seltzer et al. 2020), anti-seizure (Devinsky et al. 2017; Izquierdo et al. 1973) and anxiolytic (Resstel et al. 2009) effects. In Australia, the UK, EU and the USA, cannabidiol has only been approved by the regulatory authorities as an adjunct therapy for use in two rare forms of intractable childhood epilepsy (Arzimanoglou et al*.* 2020). It is also used in some countries combined with everolimus for control of seizures in tuberous sclerosis (Schubert-Bast and Strzelczyk 2021). In recent years, there has been a substantial increase in the use of cannabidiol and various poorly characterised cannabis products). In the EU, cannabis-containing products (e.g. food, skin care, oils) are readily available (Hughes et al. 2022). The increase in cannabis formulations has been especially significant in countries such as Canada and the USA, where such products can now be obtained legally in some states (Weinberger et al. 2022). Much of this is for recreational use but some is for unregulated and unproven treatment of conditions that seem to be unresponsive to standard treatment (National Academies of Sciences, Engineering, and Medicine 2017). In spite of its widespread use, very little is known about how much cannabidiol can enter the brain, especially during development.

Using the rat as an animal model, we have estimated the entry of radiolabelled cannabidiol into the brain and cerebrospinal fluid (CSF) of fetal, postnatal and adult animals. As an indicator of placental transfer following maternal administration, we have compared levels of cannabidiol in the maternal and fetal circulations. Because binding of drugs by plasma proteins is thought to be an important determinant of their tissue distribution, including the brain (Qiu et al. 2023), we have also determined cannabidiol plasma protein bound and unbound fractions at different ages and established that although albumin is the main protein binding the drug, this is most likely due to its relative abundance rather than greater affinity. As the main mechanism of action of cannabidiol is via interaction with the endocannabidiol system, as well as binding to specific central and peripheral receptors (Peng et al. 2022), we have mined available rat RNA seq datasets of fetal, postnatal and adult brain and choroid plexus as well as E19 placenta (Koehn et al. 2020) to determine developmental changes in the expression of 13 main cannabidiol receptors.

## Methods

### Ethical statement

All animal experimentation procedures were approved by the Alfred Research Alliance Animal Ethics Committee (Ethics ID: E/8357/2023/M). Experiments were conducted in compliance with Australian National Health and Medical Research Guidelines. Animals were supplied by the Monash Animal Research Platform (MARP) and allowed a minimum of 2 days in a stress-free environment in the Alfred Medical Research and Educational Precinct Animal Services before use. Before the start of experiments, all animals were assessed by the Animal House staff for their health status. All surgeries were short term and were conducted under deep terminal anaesthesia (see below). Only experienced researchers handled the animals. Every effort was made to minimise stress and suffering. All aspects of the study conformed to the ARRIVE guidelines.

### Animals

The Sprague–Dawley (RRID: RGD_728193) strain of *Rattus norvegicus* was used. They were supplied by the Monash University Clayton Campus Animal Facility. After transfer to the Alfred Medical Research and Educational Precinct Animal Services, time-mated pregnant rats were kept singly in a Microenvironmental Isolation Containment System cages (38.5 × 40 × 24 cm on S-Select grade premium scientific aspen wood chip bedding and paper wool nesting material made from virgin paper with no added chemicals) on a 12-h light/dark cycle with ad libitum access to food (irradiated rat diet dry pellets of a fixed formulation for rats, Speciality Feeds, Western Australia) and water. Pregnant rats were used at embryonic day E19 or allowed to litter and their offspring used at postnatal day P4 and P12. Non-pregnant female rats were also used. For all age groups, animals came from at least two separate litters. Numbers of rats used at each age are shown in Table 1.

**Table 1:** Numbers of animals used at different ages in different experiments

**Age**	**Route of drug administration**	**Number of animals (*n*)**	**Mean weight (g) ± SD**	**Mean crown-rump length (cm) ± SD**
E19	*i.p.* to fetus	11 (*2 litters) 8 female, 3 male	2 ± 0.2	3 ± 0.2
	*i.v.* to dam	18 (*3 litters) 10 female, 8 male	2 ± 0.3	3.2 ± 0.1
P4	*i.p*	10 (4 litters) 5 female, 5 male	10 ± 2	nd
P12	*i.p*	5 (2 litters) 3 female, 2 male	29 ± 5	nd
Adult (non-pregnant)	*i.p*	3 3 female, 0 male	202 ± 2	nd
Adult (pregnant)	*i.v*	3 3 female, 0 male	337 ± 31	nd

Body weight, mean ± SD. Crown-rump length, mean ± SD. *From E19 pregnant dams; *nd* not determined, *i.p.* intraperitoneal, *i.v.* intravenous

The time-mated females were all primigravida. E0 was taken as the day a vaginal plug was identified and P0 as the day of birth. E19 is a fetal stage of development when adequate volumes of blood and cerebrospinal fluid (CSF) can be obtained for analysis (Dziegielewska et al. 1981) and large enough to permit *i.p*. injections (Koehn et al. 2019a). Also, individual fetuses, while still inside the uterine horn, can be injected intraperitoneally (*i.p.*) and kept in a viable state for different periods of time (Koehn et al. 2019a, 2020; Huang et al. 2023). P4 is a stage of brain development equivalent to that of very premature but viable human infants of 22–24 weeks gestation (Clancy et al. 2001; Workman et al. 2013). These ages have been used in earlier studies of other drugs which allows comparisons to be made for different drugs (Koehn et al. 2019a, 2020; Toll et al. 2021; Huang et al. 2023). Animal numbers (Table 1) were based on previous experiments; they were the minimum number required to detect a significant difference between groups at *p* < 0.05. All fetuses and pups were sexed and equal numbers of male and female were used when possible, so that potential differences in drug entry due to sex could also be analysed (Table 1). The rats were allocated to experiments by the Animal House staff, who had no knowledge of the particular experiments to be performed; experimenters were blinded to this allocation to avoid selection bias. All experiments were conducted between 8am and 4 pm.

### Drug injectate preparation

Cannabidiol doses were based on those used in clinical practice (Australian Medicines Handbook, 2021) and adjusted for animals’ body weight (10 mg/kg)**.** All cannabidiol (Sigma-Aldrich, C7515) doses were dissolved in 52% ethanol/sterile saline solution (0.9% NaCl). Volumes administered to animals were calculated so that the blood alcohol level would remain as low as possible and well below the lethal dose for each age (Wiberg et al. 1970, 1971). A radiolabelled [^3^H]-cannabidiol tracer (Moravek, US, MT2154) was added to the injectate at 1 μCi (E19), 2 μCi (P4), 4 μCi (P12), 4 μCi (adult non-pregnant) or 25 μCi (adult pregnant).

### Anaesthesia, experimental procedure and sample collection

Two injection protocols were used: (i) intravenous (*i.v.*) injection of cannabidiol to pregnant dams at E19 to estimate transfer across the placenta and fetal brain entry over a period of up to 100 min, and (ii) intraperitoneal (*i.p.*) injection to individual animals at all ages to estimate cannabidiol entry into CSF and brain at 30 min after injection.

#### Intravenous injection of cannabidiol into E19 pregnant dams

Pregnant dams at E19 were anaesthetised with *i.p.* urethane (25% w/v urethane, Sigma, 1 ml/100 g body weight). A deep level of anaesthesia was assessed by the absence of pedal withdrawal reflex as outlined in the National Health and Medical Research Council (2008). The rats were placed supine on a temperature-controlled heating pad (38.5 °C). A tracheal catheter was inserted to maintain a clear airway. A catheter was inserted into the femoral artery; this was used for time-matched maternal arterial blood sampling. After each arterial blood sample was collected, the cannula was flushed with 0.5 ml heparinised saline (Hospira Inc, 5000 units/ml). A single dose of 10 mg/kg cannabidiol containing traces of [^3^H]-labelled cannabidiol (25 µCi per dam) was injected directly into the femoral vein using a fine needle (BD Ultra-Fine Insulin Syringe). Blood, cisternal CSF and brain tissue samples were taken from each fetus together with time-matched maternal blood sample as previously described for other drugs (Koehn et al. 2019a; Chiou et al. 2021; Toll et al. 2021; Huang et al. 2023). Blood from the fetuses was collected using heparinised glass micropipettes and CSF collected using a fine glass micropipette. Brain samples from two sites were collected: (i) cortical samples from the frontal/parietal lobes dorsal to the lateral ventricles (as described previously, Koehn et al. 2019b) and (ii) the brainstem. All E19 fetuses had a confirmed heart beat before sample collection. The experiment was terminated by taking a final maternal blood sample (exsanguination) from the right cardiac ventricle using heparinised syringe and a 20-gauge needle (Terumo). Following terminal exsanguination, maternal CSF samples were taken using glass micropipettes and corresponding brain samples were collected.

One pregnant E19 dam died before the completion of the experiment due to anaesthetic overdose. However, fetuses from this dam were still alive upon collection, with presence of heart beat confirmed in all fetuses; therefore, samples from these fetuses were used for fetal brain and CSF cannabidiol entry but not for the placental transfer estimations.

#### Intraperitoneal injection of cannabidiol

##### E19 fetuses

The dams at E19 were anaesthetised and prepared for experiments as described in the previous section. Fetuses were exteriorised sequentially, still within their amniotic sacs, and a single dose of 10 mg/kg cannabidiol traced with [^3^H]-labelled cannabidiol (1 µCi) was administered by *i.p.* injection to each fetus. Fetal samples were collected 30 min post-injection. The viability of each fetus was assessed at the time of sampling by checking the colour of the umbilical vessels (marked difference in colour between the veins and arteries) and presence of heart beat. A large volume of blood was taken from the right ventricle and experiment terminated by exsanguination. Cisternal CSF and brain samples were then taken from each fetus as described above and previously (Koehn et al. 2019a; Chiou et al. 2021; Toll et al. 2021; Huang et al. 2023).

##### Postnatal animals, P4, P12 and adult non-pregnant females

Animals were anaesthetised with *i.p.* urethane (2.0–2.5 g/kg, 25% w/v in sterile water). A single dose of 10 mg/kg cannabidiol containing traces of [^3^H]-labelled cannabidiol (2 µCi P4, 4 µCi P12 and adults) was administered by *i.p.* injection to all animals. After 30 min, the experiment was terminated by exsanguination from the right ventricle and blood collected. CSF and brain sampling was performed as described for E19 fetuses in the previous section.

#### Dose dependence and time course of cannabidiol entry into brain and CSF

P12 pups were anaesthetised as described above. Individual pups were injected *i.p.* with increasing doses of cannabidiol (0.1, 1.0, 10, 50 mg/kg traced with 4 µCi [^3^H]-labelled cannabidiol added). After 30 min, samples of brain and CSF were obtained as described above. The time course of entry was measured in P12 pups. Animals were anaesthetised as described in the previous section and injected *i.p.* with 10 mg/kg cannabidiol traced with [^3^H]-labelled cannabidiol (4 µCi). Individual pups were collected at 15 min, 30 min, 60 min and 120 min for sampling of the brain and CSF as described above.

### Liquid scintillation counting

Blood, CSF and tissue samples were processed immediately after collection. Plasma was separated from whole heparinised blood by centrifugation (5000 × g, 2 min). CSF samples were centrifuged (5000 × g, 2 min) and microscopically examined for traces of red blood cell contamination (Habgood et al. 1992). Any contaminated samples would be rejected but none was detected. A sample of injectate was measured to confirm uniformity of injected material. Samples were weighed and transferred to scintillation vials. Brain samples were solubilised in 0.5 ml Soluene350 (PerkinElmer) overnight at 36 °C. The strongly alkaline Soluene350 was neutralised with glacial acetic acid (2 drops, Sigma). Samples were mixed with 3.6 ml scintillant (Emulsifier-safe, PerkinElmer) and each sample counted for 5 min in a liquid scintillation counter (Tri-Carb 4910 TR, PerkinElmer) with luminescence correction activated. Radioactivity was expressed as disintegrations per minute (DPM) calculated from counts per minute. Blank samples containing the same tissues with no radioactivity were run at the same time to establish the background counts. These were always subtracted from sample counts. This method has been described in several previous studies (Koehn et al. 2019a; Toll et al. 2021; Huang et al. 2023). Radioactivity in samples was expressed as DPM/µl of plasma and CSF or DPM/mg for brain cortex and brainstem. The following equations were used to indicate entry of cannabidiol into the brain cortex, brainstem and CSF (Eq. 1) or its placental transfer (Eq. 2).

1 $$\mathrm{Brain\;or\;CSF\;entry}\;=\;\frac{\mathrm{Cortex,\;brainstem\;or\;CSF\;DPM}/\mu\mathrm{l\;or\;mg}}{\mathrm{plasma\;DPM}/\mu\mathrm{l}}\;\times\;100\%$$

2 $$\begin{array}{c}\mathrm{Placental\;transfer}\;=\;\frac{\mathrm{Fetal\;plasma\;at\;time}\;x\;\left(\mathrm{DPM}/\mu\mathrm{l}\right)}{\mathrm{Average\;maternal\;plasma\;up\;to\;time}\;x\;\left(\mathrm{DPM}/\mu\mathrm{l}\right)}\;\times\;100\%\\\qquad\qquad\mathrm x\;=\;\mathrm{fetal\;sampling\;time}\end {array}$$

These ratios are a standard method used because of unavoidable variations in the plasma concentration between individual experiments (Davson and Segal 1996). The level of [^3^H]-cannabidiol in brain and plasma samples represents its accumulation at the end of each experimental period (in most experiments 30 min). This reflects a combination of entry, retention and removal and will be referred to as a measure of entry but not as the rate of entry, which would require a different experimental design.

### Determination of total protein concentration

Pierce™ Bicinchoninic Acid (BCA) Protein Assay Kit (Thermo Scientific) was used to determine total protein concentration of samples used in plasma protein binding studies. Manufacturer’s protocol was followed. Briefly, stored plasma samples were thawed at room temperature and diluted in phosphate buffer solution (Gibco, Dulbecco’s Phosphate Buffer Saline Powder). Numbers of animals from which plasma samples were used at different ages are shown in Table 2.

**Table 2:** Plasma samples used for total protein measurements

**Age**	***n***
E19	7
P4	5
P12	6
Adult (non-pregnant)	4
Adult (pregnant)	7

*n* refers to number of samples from individual animals. At E19 and P4 plasma, samples were pooled from multiple animals

Albumin standards (bovine serum in 0.9% saline and 0.05% sodium azide) were diluted in phosphate buffer solution as specified by manufacturer instructions. Twenty-five microlitres of each sample in duplicate and albumin standards were loaded into a 96-well flat bottom plate (Costar) before 200 µl of BCA working reagent (50:1 Reagent A, B) was added to each well and left to incubate at 37 °C for 30 min. The plate was analysed using absorbance imaging (BMG Labtech Fluostar Optima). Results were read from the standard curve derived from the albumin standards and multiplied by the initial dilution factor.

### Plasma protein binding of cannabidiol

Binding of cannabidiol to protein in blood plasma was determined using two approaches (ex vivo and in vitro), based on a previously established method of ultrafiltration using 30 kDa molecular weight cut-off centrifugal filters (Centrifree^®^, Merck Millipore, Qiu et al. 2023). Additionally, a novel method based on polyacrylamide gel separation and incubation was also developed to identify which plasma proteins might be the main cannabidiol binding fraction in circulation.

#### Ex vivo

Plasma samples used in this method were the terminal blood samples collected from animals that were used for in vivo experiments described above. They were from E19 fetuses, P4 and P12 pups, and non-pregnant adult females (Table 3). Blood was collected into heparinised tubes and plasma separated immediately by centrifugation at 5000 × g for 2 min. Samples of 200 μl of plasma from individual animals or pooled samples (Table 3) were collected. A 10 µl aliquot of plasma was removed as a reference point and the rest of each sample was aliquoted into centrifugal filters and centrifuged until at least 5 µl of protein-free filtrate was separated and collected. Adult samples were spun for 2.5 min, while samples from fetuses and pups were spun for 1.5 min. Plasma volume reduction during the process of ultrafiltration has been shown to negligibly affect the binding equilibrium for some drugs, with the concentration of free drug in the filtrate constant up to ~ 40% of the sample volume filtered (Whitlam and Brown 1981). The filtrate (free fraction) together with whole plasma protein fraction was collected for liquid scintillation counting (Tri-Carb 4910 TR, PerkinElmer, Inc.) and the cannabidiol free fraction (%) was determined using Eq. 3.

**Table 3:** Plasma samples for ex vivo cannabidiol binding

**Age**	***n***
E19	3 (pooled)
P4	4 (pooled)
P12	5 (3 female, 2 male)
Adult (non-pregnant)	3

At P12 and adult, plasma from individual animals was used; at E19 and P4, samples were pooled from several fetuses or pups to obtain adequate sample volumes (minimum 200 μl); *n* is number of individual samples/animals

3 $$\mathrm{Free\;Fraction\;ratio}\,\left(\%\right)\;=\;\frac{\mathrm{Protein\;free\;fraction\;DPM}/\mu\mathrm{l}}{\mathrm{Whole\;plasma\;protein\;fraction\;DPM}/\mu\mathrm{l}}\;\times\;100\%$$

#### In vitro

This method has been described before (Qiu et al. 2023). Briefly, control, untreated time-mated pregnant females at E19, E19 fetuses, P4 pups, P12 pups and non-pregnant adult females were deeply anaesthetised via *i.p.* injections of urethane (Sigma Aldrich: 25% w/v, 1 ml per 100 g of body weight). Blood was collected by terminal cardiopuncture into heparinised tubes and plasma separated immediately by centrifugation at 5000 × g for 2 min. Separated plasma was stored at − 20 °C until use. As stored plasma samples can become more alkaline over time, pH was always adjusted back to ~ 7.4 by addition of 1 μl/ml of 2.5 M hydrochloric acid (Sigma) and confirmed by pH indicator strips (Merck, Qiu et al. 2023). Samples from at least 3 individual animals were collected for each age. At E19, plasma was pooled from multiple fetuses from each litter and at P4 some plasma samples were also pooled due to small volumes of blood obtained (Table 4). Processing of samples was identical to the ex vivo method described above.

**Table 4:** Plasma samples used for in vitro determination of cannabidiol protein binding

**Age**	***n***	**Sex**
E19	5 (pooled)	nd
P4	3 (pooled)	nd
	3 (individual)	1 female, 2 male
P12	6	1 female, 5 male
Adult (non-pregnant)	5	5 female
Adult (pregnant)	7	7 female

At P12 and adult, plasma from individual animals was used; at P4, three samples were pooled and three were from individual animals; at E19, samples from several fetuses were pooled to obtain adequate volumes (minimum 200 μl per sample). *n* is number of individual samples/animals; *nd* not determined

### Separation of plasma proteins by polyacrylamide gel electrophoresis

To identify which plasma proteins bind cannabidiol, analysis of binding was done using a novel method combining polyacrylamide gel electrophoresis (PAGE) with liquid scintillation counting. Numbers of animals from which plasma samples were used (see Table 5).

**Table 5:** Plasma samples used for gel electrophoresis and cannabidiol incubation

**Age**	***n***
E19	1 (pooled)
P4	2
P12	2
Adult (non-pregnant)	3

*n* refers to number of individual animals/samples. At E19, plasma sample was pooled from multiple animals

Loading buffer (LB, Chem Supply) with dithiothreitol (DTT 0.1 M, Invitrogen) was added to plasma samples at a ratio of 1:5, LB:plasma, before the sample was finally diluted in saline (0.9% NaCl) in order to obtain 1:10 dilution of the plasma. The samples were reduced at 100 °C for 5 min. Plasma samples and a protein ruler (250 kDa, Thermoscientific Page Ruler Plus) were separated on a 10% acrylamide (BioRad) SDS-PAGE gel at 140 V, 300 A for 1.5 h. After separation, the gel was washed with Triton X-100 (Merck, 2.5%) in phosphate buffer solution (Gibco, Dulbecco’s Phosphate Buffer Saline Powder) 3 × 20 min for a total of 1 h. A sample portion of the gel was removed for Coomassie (Sigma Aldrich) staining, while the remainder of the gel was incubated with cannabidiol (Sigma Aldrich, 100 mg/kg) in 15 ml phosphate buffer solution with [^3^H]-cannabidiol overnight at 37 °C. After 10–12 h, the gel was washed 3 times in 15 ml phosphate buffer solution for 20 min each.

The gel was next cut into 5 mm sections, including a control section containing no samples, and each section placed into pre-weighed scintillation vials and weight recorded. A total of 3.6 ml scintillation fluid (Emulsifier-safe, PerkinElmer) was added to each vial before the samples were counted in a liquid scintillation counter (Tri-Carb 4910 TR, PerkinElmer Inc.) for 5 min. Results are expressed as a DPM/mg for gel samples calculated using Eq. 4:

4 $$\mathrm{Cannabiodiol\;Bound\;Portion}\;\left(\mathrm{DPM}/\mathrm{mg}\right)\;=\;\frac{\mathrm{Plasma\;Gel}\;\left(\mathrm{DPM}\right)}{\mathrm{weight}\;\left(\mathrm{mg}\right)}-\frac{\mathrm{Control\;Gel}\;\left(\mathrm{DPM}\right)}{\mathrm{weight}\;\left(\mathrm{mg}\right)}$$

### Expression of cannabidiol receptors

Thirteen receptors and their transcript variants (20 in total), suggested to be involved in cannabidiol binding (Peng et al. 2022; Elmes et al. 2015; Mecha et al. 2013; Table 6), were chosen to investigate any age-related changes in the brain, choroid plexus and placenta. Rat RNA-seq datasets were obtained from a previous study (Koehn et al. 2020, 2021) and datasets included placenta, brain and choroid plexus from E19 fetuses, and brain and choroid plexus from P5 pups and adults (males and females; *n* = 4 at each age). The datasets were analysed using 3 independent RNA-sequencing differential expression analysis programmes (EdgeR, limma and DESeq2; Koehn et al. 2020, 2021) and comparisons were deemed statistically significant if *p*-adj < 0.05 in at least two of the three analysis tools (updated data available at: https://identifiers.org/ncbi/bioproject:PRJNA633629).

**Table 6:** List of cannabidiol binding receptor transcript variant names

**Gene ID**	**Symbol**	**Name**
NM_012784	*Cnr1*	Cannabinoid receptor type 1
NM_020543	*Cnr2*	Cannabinoid receptor type 2 (transcript variant 3)
NM_001164142	*Cnr2*	Cannabinoid receptor type 2 (transcript variant 2)
NM_001164143	*Cnr2*	Cannabinoid receptor type 2 (transcript variant 1)
NM_012556	*Fabp1*	Fatty acid binding protein
NM_031982	*Trpv1*	Transient receptor potential vanilloid 1
NM_001270798	*Trpv2*	Transient receptor potential vanilloid 2 (transcript variant 3)
NM_001270797	*Trpv2*	Transient receptor potential vanilloid 2 (transcript variant 2)
NM_017207	*Trpv2*	Transient receptor potential vanilloid 2 (transcript variant 1)
NM_001025757	*Trpv3*	Transient receptor potential vanilloid 3
NM_023970	*Trpv4*	Transient receptor potential vanilloid 4
NM_134371	*Trpm8*	Transient receptor potential melastatin 8
NM_207608	*Trpa1*	Transient receptor potential ankyrin 1
NM_053294	*Adora2a*	Adenosine A2A receptor A2AR
NM_001357942	*Adora2a*	Adenosine A2A receptor uORF5
NM_012585	*Htr1a*	Serotonin 1A receptor
NM_001145367	*Pparg*	Peroxisome proliferator-activated receptor gamma (transcript variant 3)
NM_001145366	*Pparg*	Peroxisome proliferator-activated receptor gamma (transcript variant 2)
NM_013124	*Pparg*	Peroxisome proliferator-activated receptor gamma (transcript variant 1)
NM_012547	*Drd2*	Dopamine-2

Gene IDs and symbols from NCBI Reference Sequences (RefSeq, O’Leary et al. 2016)

### Statistical analysis

Data from all experiments are expressed as mean ± standard deviation (SD). Differences between means were calculated for statistical significance using one-way ANOVA or two-way ANOVA with Tukey’s post hoc test for multiple comparisons (GraphPad Prism 9). Correlation between two variables was determined using two-tailed Pearson’s correlation coefficient calculation (Graphpad Prism9).

## Results

### Dose dependence and time course of cannabidiol entry into brain and CSF

**Fig. 1:**
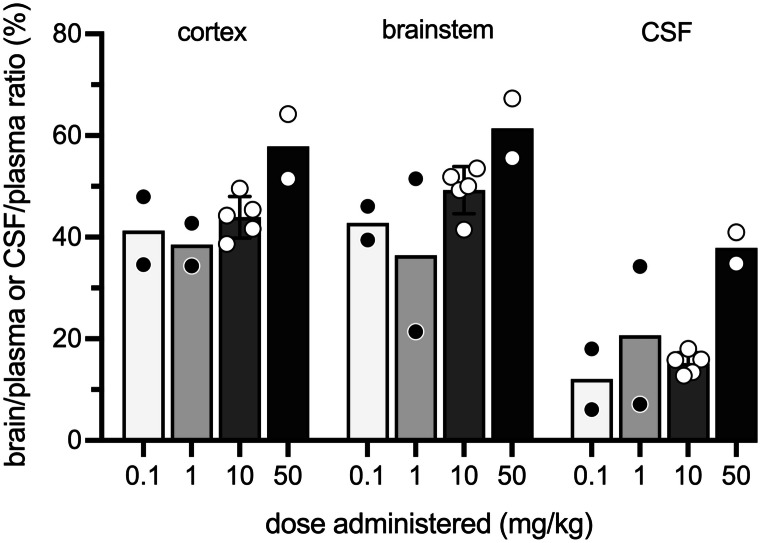
Dose response of entry of cannabidiol into brain and CSF of postnatal P12 pups. The pups were injected *i.p.* with 0.1, 1.0, 10, or 50 mg/kg, cannabidiol and samples taken 30 min post-injection. Results are expressed as ratios of DPM in brain or CSF to plasma as a %. Each point represents a single animal; *n* = 2–5. Numerical values are in Supplementary Table 1

In order to establish the effects of different doses and time of exposure of *i.p.*-administered cannabidiol, a series of experiments were performed at P12. Doses used ranged from 0.1 to 50 mg/kg of cannabidiol with its [^3^H]-cannabidiol tracer, adjusted to the weight of the pup. Results showed that doses of 0.1 mg/kg, 1 mg/kg and 10 mg/kg all achieved similar brain and CSF entry of around 40% brain/plasma and 10–20% CSF/plasma ratios (Fig. 1). However, when the dose was increased to 50 mg/kg, there appeared to be a greater entry into the brain, to nearly 60% and CSF to nearly 40% (Fig. 1). Numerical data are provided in Supplementary Table 1.

**Fig. 2:**
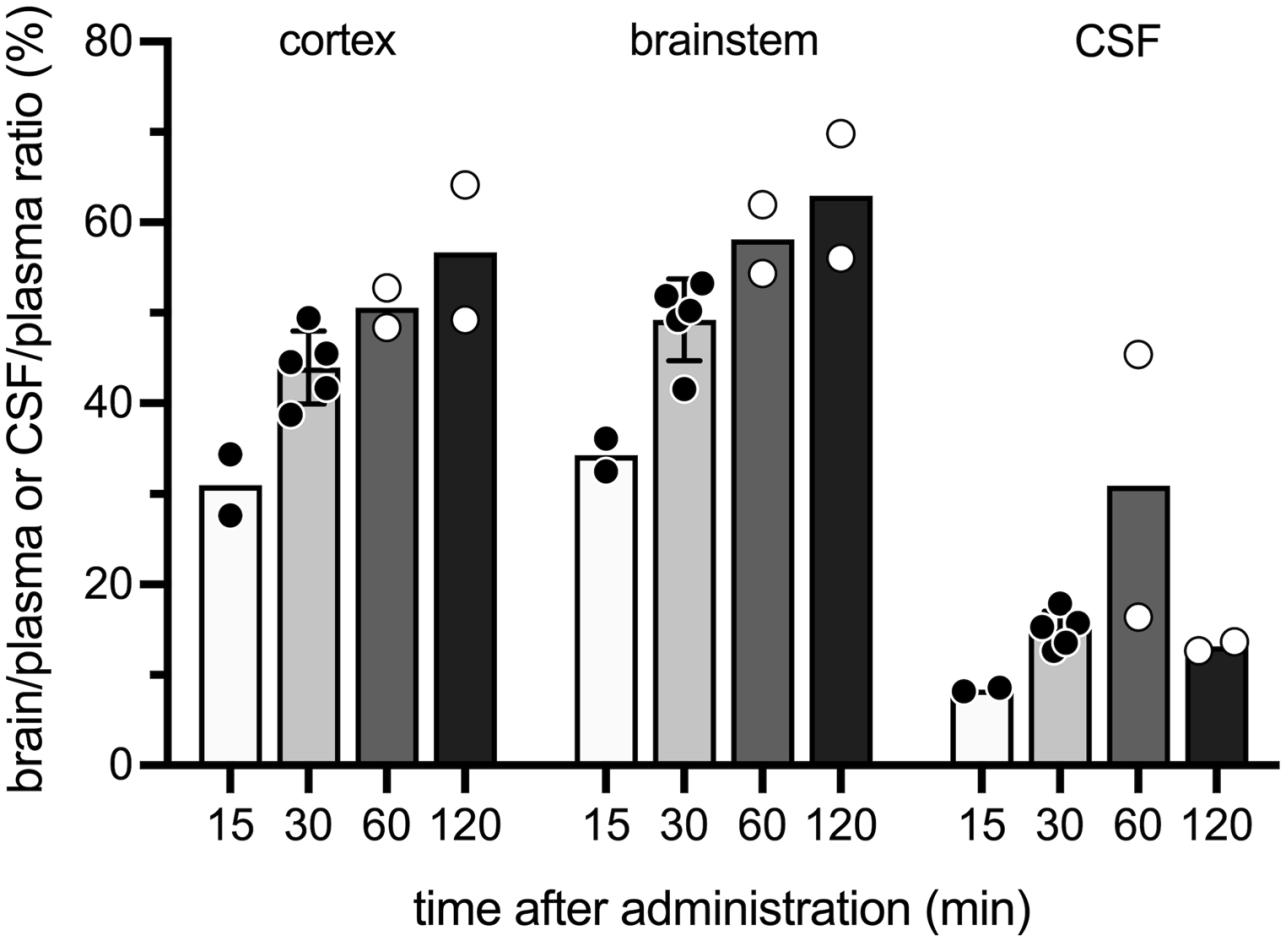
Time course of entry of cannabidiol into brain and CSF of P12 pups. Pups were injected *i.p.* with 10 mg/kg, cannabidiol and samples collected from 15 min post-injection. Results are expressed as ratios of DPM in brain or CSF to plasma (%). Each point represents a single animal; *n* = 2–5. Numerical values are in Supplementary Table 2

For the time course, the dose of 10 mg/kg of cannabidiol with its radiolabelled tracer was injected *i.p.* into P12 rat pups and allowed to circulate for 15, 30, 60 and 120 min before plasma, CSF and brain tissue collection. As shown in Fig. 2, entry of cannabidiol (expressed as brain or CSF/plasma ratios %) appeared to increase overtime, from around 30% at 15 min to around 60% at 120 min. The rate of increase was largest within the first 30 min, with little difference between 30 and 60 min (Fig. 2). As cannabidiol has been shown to be extensively metabolised within the first hour following an *i.p.* injection in guinea pigs (Cabral-Pereira et al. 2020), a time point of 30 min was chosen. Numerical data are provided in Supplementary Table 2.

### Transfer across the placenta and entry into fetal brain and CSF of maternally administered cannabidiol

#### Placental transfer of cannabidiol

**Fig. 3:**
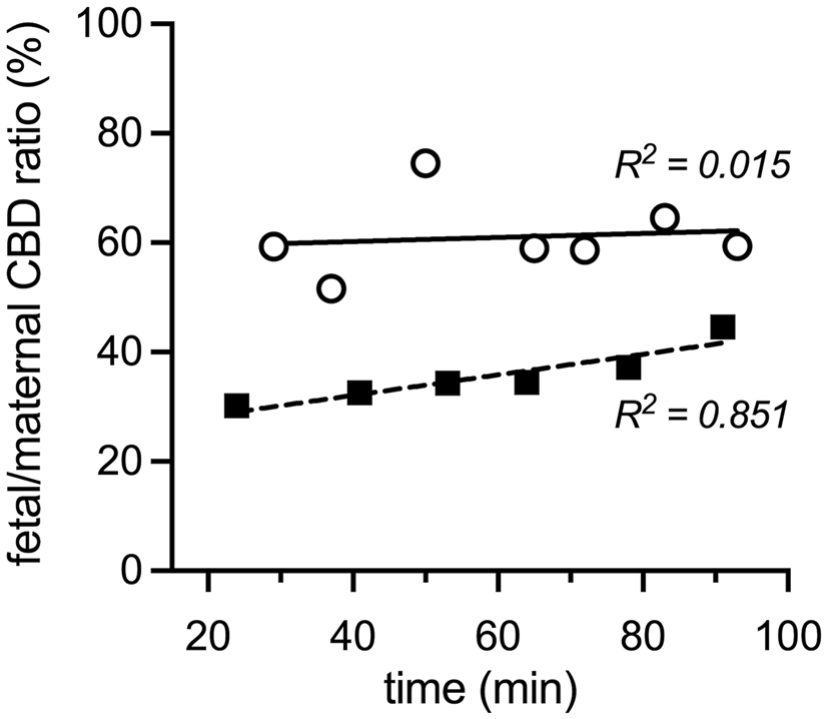
Placental transfer of cannabidiol (CBD) from maternal to fetal circulation. Results are expressed as fetal/maternal plasma DPM ratios (%). E19 dams were administered cannabidiol *i.v.*, 10 mg/kg. Each point represents a single fetal sample time-matched with a maternal sample. *n* = 2 dams and 13 fetuses, see Table 1. *R*^2^ of each line refers to the Pearson correlation coefficient

Pregnant E19 dams were injected *i.v.* with a clinical dose of cannabidiol (10 mg/kg) including [^3^H]-cannabidiol tracer. The data on fetal/maternal plasma cannabidiol ratio (%) over time in two dams that were administered the drug are shown in Fig. 3. In one dam, the ratio (around 60%) was stable for the duration of the experiment. In the other dam, there was a statistically significant positive correlation between transfer of cannabidiol and time (*R*^2^ = 0.84; Fig. 3). The mean fetal/maternal plasma ratio for dam 1 was 61 $\pm 7\mathrm{\%}$, and dam 2 was 36 $\pm 5\mathrm{\%}$, with a combined mean value of 49 $\pm 14\mathrm{\%}.$ These data show that the placenta is able to substantially restrict the movement of cannabidiol from the mother to the fetus.

#### Entry of cannabidiol into E19 fetal brain and CSF

**Fig. 4:**
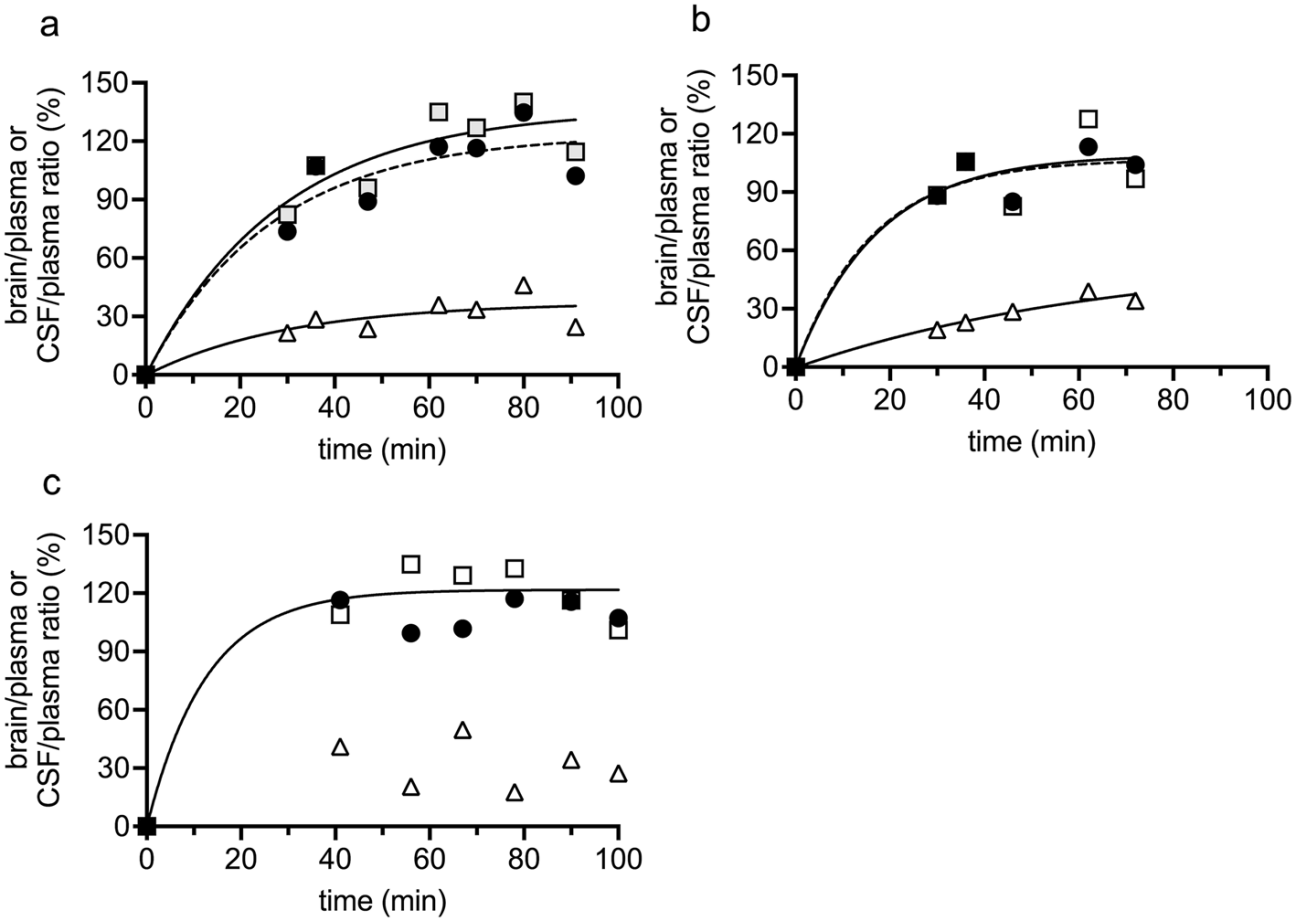
Entry of cannabidiol from fetal plasma into fetal cortex (circle), brainstem (square) and CSF (triangle). Data for three E19 pregnant dams shown, at **a**, **b** and **c**, each injected with cannabidiol *i.v.* 10 mg/kg. Fetal sampling from 30 min post-injection up to 100 min. Each point represents a single fetus. Numerical data in Supplementary Table 3. Curves of best fit determined using non-linear least squares regression analysis, Graphpad Prism 9. In **c**, it was not possible to fit a non-linear curve for cortex and CSF due to the distribution of data

Figure 4 illustrates cannabidiol entry into fetal brain (cortex and brainstem) and CSF expressed as ratios relative to plasma (%) over the duration of the experiment. At the earliest collection time (30 min), brain/plasma ratio (cortex and brainstem combined) in the fetuses from the first dam was 78% and from the second dam was 89% (Fig. 4a, b and Supplementary Table 3). In the third dam, the earliest sample was collected at 40 min, where the entry of cannabidiol was already over 100% (Fig. 4c). The highest ratio obtained in all experiments was between 60 and 80 min (137%, 121% and 125% respectively), declining to 108%, 101% and 104% respectively by the end of the experiment (up to 100 min, Supplementary Table 3).

Table 7 shows the aggregated data from Fig. 4 for brain/plasma and CSF/plasma ratios over the 100 min of the experiment. E19 fetal brain entry ratios for cannabidiol administered *i.v.* via the dam were 105 $\pm$ 15% for brain cortex and 113 $\pm$ 19% for brainstem. For CSF, the ratio was 31 $\pm$ 9% (Table 7).

**Table 7:** Entry of cannabidiol into fetal brain and CSF at E19

**Time of collection (min post-injection)**	**Cortex *n* = 3**	**Brainstem *n* = 3**	**CSF *n* = 3**
30–40	93 ± 22	93 ± 14	28 ± 12
60–80	122 ± 11	134 ± 6	35 ± 15
End (up to 100)	105 ± 3	104 ± 9	30 ± 6
Aggregated data	105 ± 15	113 ± 19	31 ± 9

Cannabidiol was administered *i.v.* to the dam. Samples were collected at 10 min intervals up to 100 min post-injection. Values are mean $\pm$ SD for brain/plasma (%) and CSF/plasma (%). Cortex and brainstem values were not significantly different from each other at any stage (*p* > 0.4, two-way ANOVA). All CSF values were significantly lower than all brain values (*p* < 0.0001, two-way ANOVA). All numerical data in Supplementary Table 1

### Age-dependent brain and CSF entry of cannabidiol

In order to establish if brain and CSF entry of cannabidiol is age related, fetal rats at E19, postnatal rats P4, P12 and non-pregnant adult females were individually injected *i.p.* with 10 mg/kg cannabidiol traced with [^3^H]-cannabidiol as described in the “Methods” section.

#### Fetuses at E19

In E19 fetuses, the overall entry of cannabidiol was 65 ± 39% into the cortex, 59 ± 26% into the brainstem and 18 $\pm$ 4% into the CSF; entry into the two brain regions was not significantly different; however, entry into the CSF was significantly lower than in cortex (*p* < 0.001) and brainstem (*p* < 0.01) as shown in Table 8.

**Table 8:** Entry of cannabidiol into brain and CSF at different ages

**Age**	***n***	**Cortex**	**Brainstem**	**CSF**
E19	11 (2 litters)	65 ± 39*	59 ± 25.7	18 ± 4
P4	10 (4 litters)	42 ± 4*	46 ± 14	13 ± 4
P12	5 (2 litters)	44 ± 4	50 ± 5	15 ± 2
Adult	3	60 ± 3	51 ± 11	30 ± 14

Cannabidiol was administered *i.p. *Samples were collected at 30 min post-injection. Values are mean $\pm$ SD brain/plasma ratios (%) and CSF/plasma ratios (%), *p < 0.05 using (two-way ANOVA compared to all other groups)

#### Postnatal pups, P4 and P12

In P4 pups, the entry of cannabidiol was 42 ± 4% into the cortex, 46 ± 14% into the brainstem and 13 ± 4% into the CSF. In P12 pups, the entry of cannabidiol into the brain and CSF was 44 ± 4% (cortex), 49 ± 5% (brainstem) and 15 ± 2% (CSF) respectively (see Table 8).

At both postnatal ages, cannabidiol entry between brain regions did not differ, whereas entry of cannabidiol was significantly lower into CSF when compared to the cortex (*p* < 0.0001), and brainstem (*p* < 0.0001).

#### Adult

**Fig. 5:**
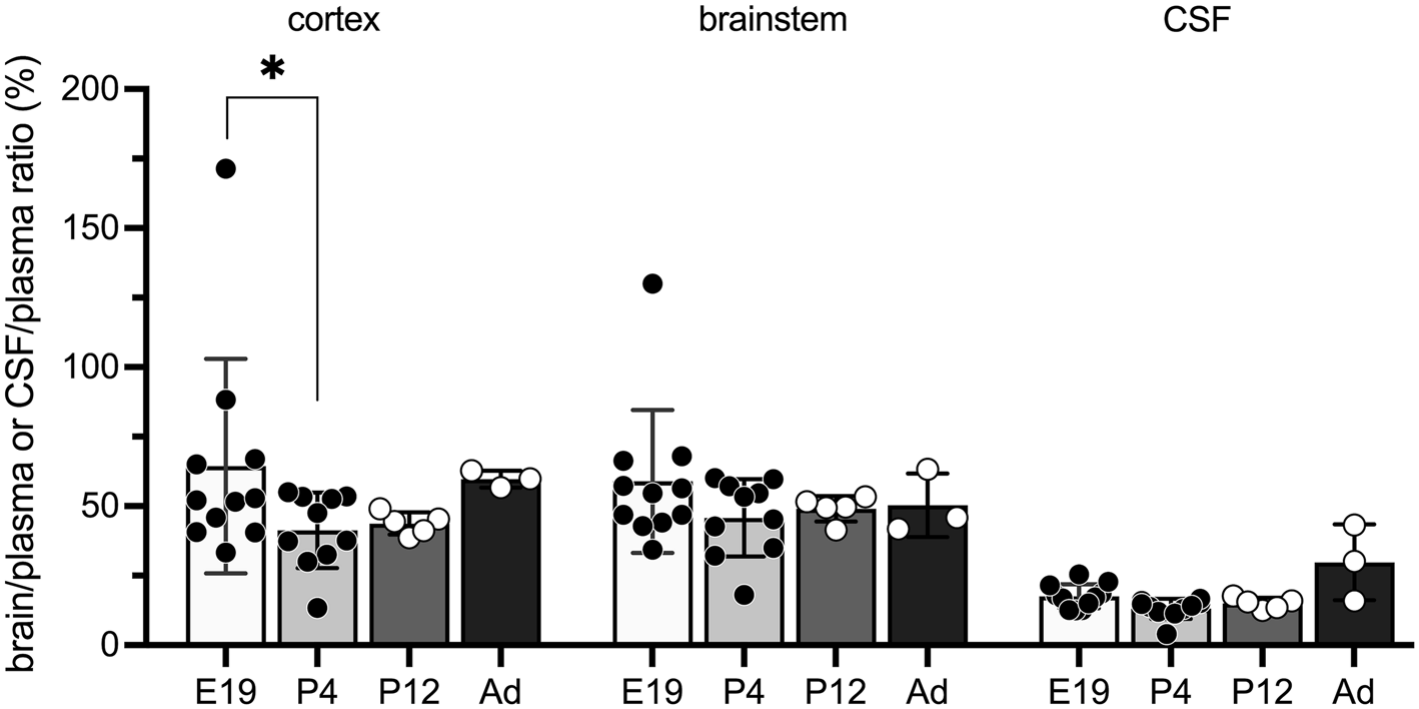
Age-dependent entry of cannabidiol into brain and CSF. Rats at E19, P4, P12 and non-pregnant adult females (Ad) were injected *i.p.* with cannabidiol, 10 mg/kg, and samples taken 30 min post-injection. Results are expressed as ratios of DPM in brain or CSF to plasma (%). Each point represents a single animal; *n* = 3–14. Mean ± SD. *p < 0.05

In non-pregnant adult female rats, entry of cannabidiol from circulation into brain cortex was 60 ± 3% and 51 ± 11% into the brainstem. Entry into the CSF was 30 ± 14% (Table 8). There was no statistical significance between the entry into the cortex and brainstem or between brainstem and CSF; however, entry into the cortex was significantly higher than into the CSF (*p* < 0.05). Figure 5 combines the results from Table 8 for entry of cannabidiol in brain and CSF at E19, P4, P12 and in non-pregnant adult female rats that were all injected *i.p.* for 30 min. There was a small but significant difference between cortex values between E19 and P4 (*p*<0.05). However, the striking findings were that (i) the brain/plasma ratios were around 50% for all ages and (ii) the CSF/plasma ratios were significantly lower (<20%) at all ages when compared to cortex. When the data were analysed in terms of animal sex, there was no significant difference in cannabidiol entry into brain and CSF in males and females at any age (see Supplementary Fig. 1).

### Plasma protein binding

Plasma protein binding of cannabidiol was estimated in blood plasma samples that were stored (in vitro) and in those collected immediately after termination of all in vivo experimentation; these are referred to as ex vivo. The results are shown in Tables 9 and 10.

**Table 9:** Binding of cannabidiol to rat plasma protein at different ages

**Age**	***n***	**Bound fraction** **(%)**	**Free fraction** **(%)**
E19	6 (pooled)	84.7 ± 3.1	15.3 ± 3.1
P4	3 (pooled) 3 (individual)	75.8 ± 10.5*	24.2 ± 10.5*
P12	6	74.1 ± 7.6*, **	25.8 ± 7.6**
Adult (pregnant)	7	86.8 ± 3.3*	13.9 ± 3.3*
Adult (lactating)	2	83.2	16.8
Adult (female)	5	86.2 ± 2.0*	13.8 ± 2.0*

Binding was estimated by an in vitro ultrafiltration method as described previously (Qiu et al. 2023). Mean $\pm$ SD. *p < 0.05, **p < 0.01 (two-way ANOVA compared to other groups)

**Table 10:** Binding of cannabidiol to rat plasma protein

**Age**	***n***	**Bound fraction** **(%)**	**Free fraction** **(%)**
E19	3 (pooled)	55.8 ± 5.6***	44.2 ± 5.6***
P4	4 (pooled)	69.1 ± 5.0***	30.9 ± 5.0***
P12	5	61.0 ± 5.0	38.0 ± 5.0
Adult (female)	3	51.5 ± 9.1*	48.5 ± 9.1*

Binding was estimated by ex vivo ultrafiltration as described in the “Methods” section. Mean ± SD. *p < 0.05, ***p < 0.001 (two-way ANOVA compared to all other groups)

At all ages, the free fraction of cannabidiol using the in vitro method was between 14 and 26% with the highest values at P4 and P12 and the lowest at E19 and in the adult (Table 9) indicating that cannabidiol is relatively highly protein bound (approximately 70–90%). There was no significant difference in the cannabidiol protein binding in adult females, whether pregnant, non-pregnant or lactating. However, at P4 and P12, cannabidiol was significantly less bound (higher free fraction) when compared to pregnant or non-pregnant adult rat plasma (*p* < 0.05 and *p* < 0.01 respectively). Cannabidiol binding in the E19 fetal plasma was lower than in postnatal pups and similar to values for adult female (Table 9).

In contrast to results obtained from in vitro methods, free fractions of cannabidiol in the ex vivo samples were generally higher and ranged between 31 and 50% with lowest values at P4 (30.9 ± 5.0%) and P12 (38.0 ± 5.0%) and highest in the adult (48.5 ± 9.1%) and E19 (44.2 ± 5.6%, Table 10). Contrasting to in vitro cannabidiol plasma protein binding, ex vivo results demonstrated significantly lower binding in E19 and adult (non-pregnant) plasma when compared to P4 plasma (*p* < 0.001 and *p* < 0.05 respectively). E19 plasma protein binding of cannabidiol was significantly lower than P12 (*p* < 0.05).

Total plasma protein concentrations were also determined at each age, with results presented in Table 11. Total plasma protein concentration was lowest in E19 fetuses (16.0 mg/ml) and increased progressively with age to reach 50.2 ± 2.4 mg/ml in adult non-pregnant female rat. The concentration in pregnant E19 dams was 41.1 ± 0.7 mg/ml, which was not significantly different from the non-pregnant animal (one-way ANOVA *p* > 0.2). However, total plasma protein concentration at developmental ages E19 and P4 was significantly lower than non-pregnant adult total plasma (*p* < 0.0001), and pregnant plasma (E19, *p* < 0.0001; P4, *p* < 0.001). P12 total plasma was significantly lower than adult (non-pregnant) (*p* < 0.01), and significantly higher than E19 total plasma (*p* < 0.01).

**Table 11:** Total plasma protein concentrations in rats at different ages

**Age**	***n***	**Concentration (mg/ml)**
Adult (non-pregnant)	4	50.2 ± 12.4
Adult (E19 pregnant)	7	41.1 ± 10.7
P12	6	31.6 ± 1.9
P4	5	20.6 ± 1.3
E19 fetus	7	16.0 ± 1.8

Plasma samples were obtained from animals used for permeability experiments. Adult refers to non-pregnant females; E19 pregnant is E19 pregnant dam. Mean ± SD, *n* refers to number of individual samples. The E19 plasma sample used was pooled from multiple fetuses

### Identification of plasma protein fraction binding cannabidiol

This was achieved by separating the proteins in whole plasma using polyacrylamide gel electrophoresis (PAGE) and then exposing the separated proteins in the gel to [^3^H]-cannabidiol. Adult (non-pregnant) P4 and P12 and fetal E19 plasma were separated and analysed for differences in binding of [^3^H]-cannabidiol across the 6.5 cm gel.

**Fig. 6:**
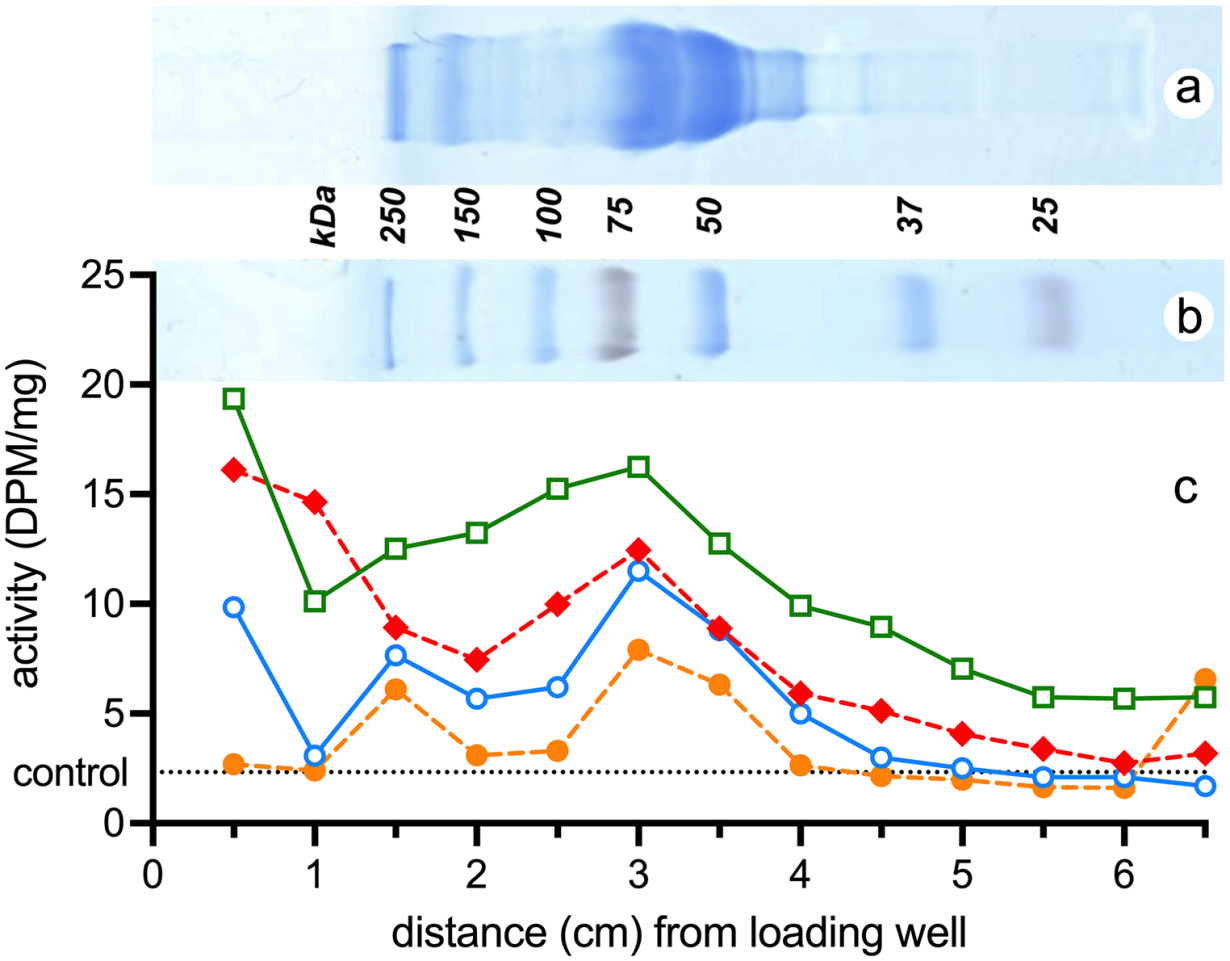
Distribution of cannabidiol binding to PAGE-separated rat plasma proteins at different ages (E19 orange circles dashed line, P4 blue circles solid line, P12 red diamonds dashed line, adult green squares solid line). Gel incubated overnight with 10 mg/kg cannabidiol traced with.^3^H-cannabidiol. Results expressed as disintegrations per minute, DPM, per mg of gel. **a** Separated plasma from E19 fetuses stained with Coomassie blue aligned to graph. **b** Separated kDa molecular standards aligned to graph. Note that the peak counts correspond to position of albumin, 66.5 kDa. The *x*-axis represents the distance (cm) of each segment from the loading well, total length of each gel was 6.5 cm. A full set of gels is presented in Supplementary Fig. 5

Duplicate gels, with additional molecular standards, were Coomassie-stained in order to visualise the spread of plasma proteins across the gel. An example of a gel of E19 separated plasma is shown in Fig. 6a and data from all ages are combined in Fig. 6b.

The highest levels of radioactivity at all ages were found in segments between 2.5 and 4 cm of the whole gel measured from the application well (Fig. 6). This corresponded to the location of the albumin fraction (66.5 kDa, Fig. 6b).

It appeared that cannabidiol overall binding increased with age (Fig. 6b) correlating with the increase in total plasma protein concentration with development (Table 11). When ratios of cannabidiol-bound counts in the albumin fraction were compared to the total counts obtained from each gel (Supplementary Table 4), the results showed that at all ages around 40% was attached to albumin (Table 12). This indicates that cannabidiol binding to albumin, although the highest of all individual plasma protein, is more likely to be a reflection of albumin abundance rather than selective affinity. All Coomassie stained gels and obtained radioactivity counts (DPM/mg) are shown in Supplementary Fig. 5 and Supplementary Table 4.

**Table 12:** Binding of cannabidiol to albumin

**Age of plasma sample**	***n***	**Albumin bound fraction (%)**
Adult (non-pregnant)	3	37.1 ± 5.2
P12	4	39.2 ± 4.4
P4	3	38.0 ± 5.9
E19	1	38

Mean percentage of cannabidiol bound to albumin fraction as a ratio of all counts recovered following PAGE separation (%). Mean ± SD, *n* refers to number of individual samples. The E19 plasma sample used was pooled from multiple fetuses

### Expression of cannabidiol receptors

**Fig. 7:**
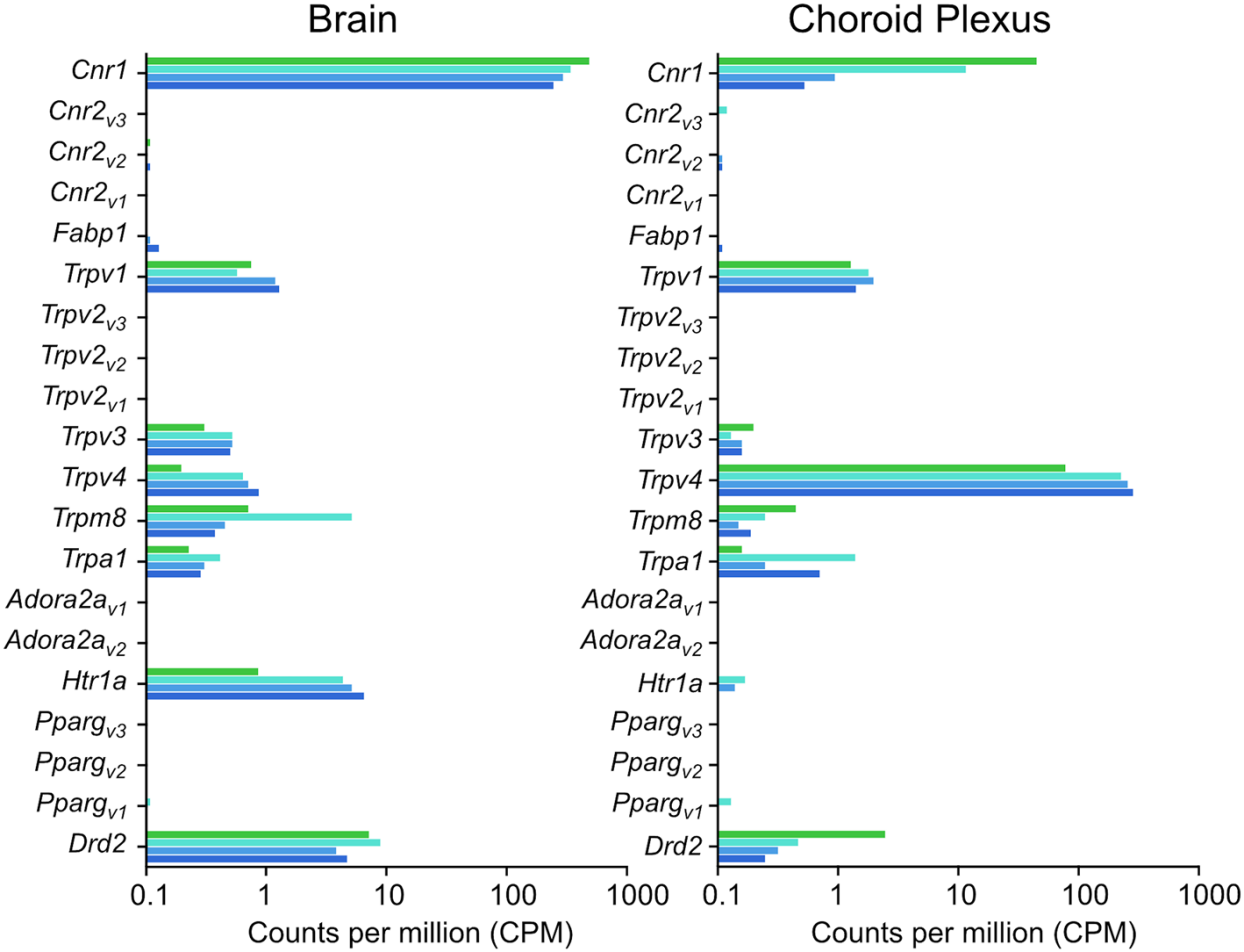
Expression of cannabidiol receptors present in control rat brain and choroid plexus at three developmental ages: E19, P5 and adult estimated from RNA-sequencing using average normalised counts per million, CPM, from EdgeR analysis. Bars are from top to bottom E19 (green), P5 (aqua), adult female (light blue) and adult male (dark blue). Counts, CPM, are displayed in Supplementary Table 5, datasets from Koehn et al. (2020, 2021). Where variants have been identified, these are shown as v1, v2 and v3, e.g. *Pparg*_*v3*_

Datasets for E19 placenta, brain and choroid plexus together with datasets from P5 and adult brain and choroid plexus (obtained from Koehn et al. 2020, 2021) were mined for known 13 cannabidiol binding receptors (see Table 6). As there are multiple transcript variants for *Cnr2* (CB2), *Trpv2*, *Adora2a* and *Pparg*, a total of 20 transcripts were identified and all CPMs are shown in Supplementary Table 5 and illustrated in Fig. 7. Only transcripts with counts more than 1 CPM in at least one biological replicate are presented in Table 13. Seven transcripts were present (> 1 CPM) in the brain, 5 in the choroid plexus and 4 in the placenta (Table 13).

**Table 13:** Normalised counts per million (CPM) of cannabidiol receptor transcripts

**Gene ID**	**Symbol**	**E19**	**P5**	**Adult**	
				**Male**	**Female**
Brain					
NM_012784	*Cnr1*	492 ± 58	344 ± 26	297 ± 27	248 ± 22
NM_031982	*Trpv1*	0.8 ± 0.3	0.6 ± 0.3	1.2 ± 0.3	1.3 ± 0.5
NM_001025757	*Trpv3*	0.3 ± 0.3	0.5 ± 0.1	0.5 ± 0.5	0.5 ± 0.3
NM_023970	*Trpv4*	0.2 ± 0.2	0.7 ± 0.3	0.7 ± 0.5	0.9 ± 0.4
NM_134371	*Trpm8*	0.7 ± 0.7	5.2 ± 1.0	0.5 ± 0.2	0.4 ± 0.1
NM_012585	*Htr1a*	0.9 ± 0.9	4.4 ± 0.2	5.2 ± 1.8	6.6 ± 2.1
NM_012547	*Drd2*	7.3 ± 5.0	9.0 ± 5.8	3.9 ± 1.3	4.8 ± 1.0
Choroid plexus					
NM_012784	*Cnr1*	45 ± 37	12 ± 9	1.0 ± 0.9	0.5 ± 0.1
NM_031982	*Trpv1*	1.3 ± 0.3	1.8 ± 0.4	2.0 ± 0.9	1.4 ± 0.4
NM_023970	*Trpv4*	79 ± 24	228 ± 18	259 ± 21	286 ± 55
NM_207608	*Trpa1*	0.2 ± 0.05	1.4 ± 0.5	0.3 ± 0.09	0.7 ± 0.1
NM_012547	*Drd2*	2.5 ± 2.7	0.5 ± 0.3	0.3 ± 0.1	0.3 ± 0.2
Placenta					
NM_031982	*Trpv1*	7.7 ± 0.9			
NM_001025757	*Trpv3*	4.6 ± 0.6			
NM_134371	*Trpm8*	1.6 ± 0.5			
NM_001145367	*Pparg*	4.4 ± 0.7			

From RNA-sequencing data. Only transcripts present at > 1 CPM in at least one biological replicate were included. Full list of transcript CPMs is in Supplementary Table 3

In the brain, the most highly expressed transcript was *Cnr1* (CB1) at E19 (492 ± 58 CPM) which then dropped to around 250 CPM in the adult, with no statistically significant difference between males and females. In contrast, the serotonin receptor *Htr1a* was expressed at a higher level in the adult (> 5 CPM) than early in development. The second highest expressed transcript at E19 and P5 was the dopamine receptor *Drd2* which appeared to be expressed at around half the level in the adult; however, due to a high degree of variation in the E19 replicates, it was only statistically significant at P5 (*p*-adj < 0.05; Table 13). *Trpm8* was the only receptor that was expressed at the highest level in P5 brain (5.2 ± 1 CPM). For the remainder of the *Trpv* receptors (*Trpv1*, *Trpv3* and *Trpv4*), they are included in Table 13 in spite of their very low mean expression. This is due to high variation between replicates, meaning that some values reached 1 CPM (Table 13).

In the choroid plexus, a total of 5 receptors were expressed at more than 1 CPM. Of these, the highest expressed was *Trpv4* which increased from 79 ± 24 CPM at E19 to over 250 CPM in both male and female adults (Table 13). *Cnr1* (CB1) was expressed at nearly tenfold higher at E19 (45 ± 37 CPM) than in the adult where it was barely detectable. Another receptor which was expressed higher in the E19 choroid plexus was *Drd2* (dopamine receptor), 2.5 ± 2.6 CPM at E19 and less than 1 CPM in the adults.

In the placenta, only 4 transcripts were detected (> 1 CPM), all 4 were expressed at a relatively low level ranging from 1.6 $\pm$ 0.5 CPM (*Trpm8*) to 7.7 $\pm$ 0.9 CPM (*Trpv1*). *Trpv3* and *Pparg* were both expressed at just over 4 CPMs (Table 13).

## Discussion

In this study, the entry of [^3^H]-cannabidiol, defined as the accumulated level at 30 min after *i.p*. injection, has been measured at E19, P4, P12 and in non-pregnant adult female rats. In pregnant rats, transplacental transfer of [^3^H]-cannabidiol was estimated by comparing the fetal and maternal plasma levels of [^3^H]-cannabidiol following *i.v*. administrations to the mother. Time-dependent accumulation in fetal brain was also measured up to 100 min. Cannabidiol binding to total and individual plasma proteins has been determined to see whether protein binding may be a factor influencing cannabidiol entry into the developing and adult brain and CSF. Expression of receptors indicated to be involved in cannabidiol binding was mined from available datasets (Koehn et al. 2020, 2021).

### Transplacental transfer

In two pregnant dams that were injected *i.v.* with the drug, the time-averaged cannabidiol fetal/maternal plasma ratios were 36% and 61% over the 100 min experimental period (Fig. 3). Although this is a substantial difference between the two experiments, it nevertheless shows that there was a functionally important restriction on cannabidiol movement across the placenta in both, thus providing the fetus with some protection against the cannabidiol in maternal circulation. This is likely to be due to the presence of efflux transporters in the placenta particularly those of the class of ATP-binding cassette (ABC) transporters. There is currently only limited information on which ABC transporters cannabidiol may be a substrate for. Most of this is from in vitro or ex vivo studies (e.g. Auzmendi et al*.* 2020 using transformed mouse heart vascular cells). More relevant to the present in vivo experiments is the study in the perfused human placenta, which showed that cannabidiol reduced the placental transfer of glyburide, a BCRP substrate and concluded that this indicated that cannabidiol is also a BCRP substrate in the human placenta (Feinshtein et al. 2013). Thus, BCRP may be at least part of the mechanism limiting cannabidiol placental transfer in our experiments. BCRP has been identified in rat placenta (Han et al. 2018; Koehn et al. 2020) where it is highly expressed.

### Brain and CSF transfer

The results from experiments in which cannabidiol was injected *i.p.* at E19, P4, P12 and in adult non-pregnant females are summarised in Fig. 5 and Table 8. At all ages, brain/plasma ratios were around 50% and the CSF/plasma ratios were even less (< 20%). The only age-related difference that was statistically significant (*p* < 0.05) was that at P4 where the cortex/plasma ratio was lower than at E19. It is possible that this is a reflection of a transient increase in an exclusion mechanism in this brain region in the early neonatal period. Further studies would be required to clarify this. Drug transfer into the brain and CSF in vivo is determined by multiple factors including protein binding, lipid solubility, drug-receptor interactions and cellular transport mechanisms. In previous studies using the same injection and sample collection protocol in developing rats as in this study, most of the drugs showed an age-related decline in brain/plasma and CSF/plasma ratios (Koehn et al. 2019a; Toll et al. 2021); this was considered to be most likely due to upregulation of ABC efflux transporters and transcriptomic analysis provided some evidence to support this conclusion (Kratzer et al. 2013; Koehn et al. 2019a, 2020, 2021). In contrast, other drugs that did not show any clear evidence of such increasing exclusion from the developing brain and CSF were lithium, which is probably transported into the brain coupled to various ion transport mechanisms (Chiou et al. 2021), ivacaftor (LogD 5.8, Qiu et al. 2021) and olanzapine (LogD 1.9–2.8, Tetko et al. 2005; Huang et al. 2023). Ivacaftor is highly lipid soluble as is cannabidiol (LogD 6.6, Ghovanloo et al. 2018). The relation between lipid solubility and protein binding and its possible contribution to apparently limiting tissue entry (accumulation of cannabidiol) is discussed below. Olanzapine’s mechanism of action is thought to be achieved primarily by antagonism of dopamine and serotonin receptors, and accordingly, it has been observed to have high binding affinity for these receptors (Thomas and Saadabadi 2022). However, its transfer across barrier interfaces appears to be affected by ABC efflux transporters such as P-glycoprotein (Boulton et al. 2002; Wang et al. 2004).

The strikingly low and unchanging CSF/plasma ratios for cannabidiol do not correlate with the much higher protein concentrations in CSF early in development (Dziegielewska et al. 1981), nor do they correlate with the increasing turnover of CSF (sink effect) during development (Saunders 1992). Therefore, it is most likely that entry of cannabidiol into the CSF is a combination of relative high lipid solubility, protein binding, CSF flow and diffusion into the brain across CSF-brain interface.

As is the case for the placenta discussed above, there is also not much information about whether cannabidiol is a substrate for ABC transporters at the blood-brain and blood-CSF barriers. One of the few in vivo studies of cannabidiol entry into the brain was in wild type, and in *bcrp* and *p-glycoprotein* knockout mice. The study showed that at the blood-brain barrier cannabidiol did not appear to be a substrate for these efflux transporters in adult mice (Brzozowska et al. 2016). These transporters are well known to be present in the adult and fetal blood-brain barrier (Kratzer et al. 2013; Møllgård et al. 2017). It is known that the most highly expressed efflux transporters in fetal rat choroid plexus are *Bcrp* and *Mrp3*, but several others have also been identified (Saunders et al. 2023) which may contribute to lower cannabidiol CSF/plasma ratios.

In experiments investigating placental transfer at E19, plasma, CSF and brain samples were obtained from the fetuses whose mothers had been injected *i.v*. These results are not directly comparable to those involving intraperitoneal (*i.p.*) injection of cannabidiol because of the much longer length of the experiment and different route of injection. However, the brain/plasma ratio at 30 min in the fetuses in two *i.v.*-injected dams was 78% and 89% (Fig. 4). In contrast, in the *i.p.*-injected fetuses, this ratio was about 60% (Fig. 5). The CSF/plasma ratio for fetuses from *i.v.*-injected dams was about 30% and < 20% for *i.p.*-injected fetuses (see Figs. 4 and 5). In the fetuses of the *i.v.*-injected mothers, the ratios continued to increase and reached a plateau of about 110% for brain and about 30% for CSF (Fig. 4). The increases in the longer *i.v*. experiments presumably reflect a continued entry of cannabidiol into brain and CSF beyond 30 min followed by a plateau most likely reflecting a steady state. However, the route of administration of cannabidiol has also been shown to affect the rate of metabolism and tissue entry of cannabidiol. Intravenous (*i.v.*) administration resulted in peak plasma concentrations immediately upon administration, followed by metabolism, in adult, pregnant and fetal animals (Ochiai et al. 2021; Xu et al. 2019; Ohlsson et al. 1986). An additional factor will be the rate of transfer across the placenta following *i.v*. injection compared to uptake into the fetal circulation following *i.p.* injection. The plateau in brain/plasma and CSF/plasma ratios represents the interaction between continued entry, receptor binding of the cannabidiol and its metabolism (see also below).

### Plasma protein binding of cannabidiol

Protein binding of drugs is traditionally measured in samples that have been collected from patients or experimental animals and stored for varying periods of time. We refer to this as in vitro measurement. It represents the equilibrium between free drug and drug bound to plasma proteins in isolation from its normal situation in the circulation. There is a wide variation in the binding of different drugs to plasma proteins measured in vitro; e.g. Zhang et al. (2012) reported that around half of 222 drugs studied showed a free fraction of about 10%. In a more limited study of 17 drugs, all were found to have free fractions between < 1 and 9%, but rat plasma showed much higher free fractions for the same drugs of up to 70% (Colclough et al. 2023). Qiu et al. (2023) reported a free fraction of 65–85% for several drugs in adult rats. However, for highly lipid soluble drugs such as ivacaftor, the free fraction is much smaller (< 1%, Qiu et al. 2023). In the present study, the free cannabidiol fraction was estimated to be around 15% (Table 9).

Most drugs are reported as binding to albumin, which is by far the quantitatively dominant protein in adult plasma (Ascoli et al. 2006). Drug binding to other plasma proteins seems to have been little studied apart from ⍺1-acid glycoprotein (Ascoli et al. 2006). In vivo drugs are in equilibrium not only with proteins in plasma but also with the different cells, tissues and organs of the body. These exchanges of drug depend on their lipid solubility, molecular size, metabolism, drug receptor interactions and transport mechanisms in the interfaces between blood and tissue. In some cases, these interfaces are quite restrictive. For example, the blood-brain barrier, where there are efflux mechanisms, particularly ABC transporters, excludes or limits entry of many drugs into the brain. In the present study, we have measured protein binding of cannabidiol in plasma samples obtained from experimental animals and processes as rapidly as possible; this we refer to as ex vivo determination. The aim was to obtain estimates of protein binding that might be a better reflection of the situation in vivo. Estimates were obtained for plasma samples from different ages of animals.

In adult female rats, pregnant and non-pregnant, the free fraction of drug was much higher (40–50%) than when measured in vitro (see Tables 9 and 10). The functional significance of the free fraction of a drug is that it is the free fraction of drug that is exchangeable with cells, tissues and organs (Wanat 2020; Summerfield et al. 2022). These results suggest that in vivo the exchangeable fraction of a drug may be much higher than is apparent from in vitro measurements. In plasma samples from the youngest animals (E19 and P4), the free fraction estimated ex vivo was lower than in postnatal pups and similar to adult values estimated in vitro (15–20%). These lower free factions are perhaps a reflection of greater protein binding by the proteins such as ⍺-fetoprotein, transferrin, ⍺_1_-acid glycoprotein and fetuin that have much higher concentrations than in adult plasma, where albumin dominates (Dziegielewska et al. 1981). However, PAGE separation of proteins in plasma from different ages indicated little binding to proteins with lower molecular size than albumin (Fig. 6).

### Expression of cannabidiol receptors

Cannabidiol binds to a wide variety of receptors in the peripheral, central and immune systems, leading to extensive changes throughout the body which are yet to be fully understood. The leading mechanism of action through which most of these changes occur is thought to be mediated by the endocannabinoid system (Mouslech and Valla 2009; Peng et al. 2022).

During development, the endocannabinoid system receptors, such as CB1, are integral to brain development during fetal stages, and initiation of nutrient intake postnatally (Fride et al. 2009). Exposure to compounds affecting the endocannabinoid system has been shown to alter neurotransmitter release and impair neurodevelopment and placental development (Rossi et al. 2023; Sarikahya et al. 2022; Chang et al. 2017). There is no information available to make a direct correlation between the expression of cannabidiol receptors and cannabidiol entry into tissues; however, it is well known that endocannabinoids and the CB1 receptor play a critical role in brain development by affecting neuronal progenitor differentiation as well as cellular migration and synapse formation (Fride et al. 2009). Therefore, it can be expected that once cannabidiol reaches the fetal brain, its action via CB1 receptor, which is expressed at a higher level than in the adult, could have significant developmental effects. On the other hand, serotonin receptor *Htr1a*, expressed at higher levels in the adult, could have more pronounced effects in the more mature brain (Fig. 7 and Table 13). This could provide an indication of the importance of cannabidiol intake during pregnancy on potential deleterious effects in both the maternal and developing brain.

## Limitations of the study

1. This study has been carried out in pregnant rats at a single gestational age (E19), two postnatal ages (P4, P12), pregnant and non-pregnant adult females. The rat and human placentas are both classed as hemochorial (Dawe et al. 2007; Furukawa et al. 2019) but there are differences in morphology, in particular that the rat placenta has three morphological layers between the fetal and maternal circulations, whereas the human as only one (Furukawa et al. 2019). However, that might mean that the changes in placental permeability might be more prominent in the human. Studies in isolated perfused human placenta would contribute to validating results from rat studies. However, these were outside the scope of this study.
2. Drug levels contained within the blood vessels of brain tissue (i.e. residual vascular space) were not taken into account in the present study. Vascular space in the rat brain from E16 to adult has been reported to be around 2–4% (Toll et al. 2021; Qiu et al. 2022). This is particularly important in experiments where entry of drugs into the brain is low. Because cannabidiol brain entry was substantial, the interpretation of results would not be expected to be affected.
3. Metabolites of cannabidiol were not measured and the drug was estimated by its radioactive label rather than actual concentration. There is evidence that measurable metabolism does not occur until after 30 min after *i.p.* administration (Cabral-Pereira et al. 2020). Most of our experiments lasted 30 min. In the longer experiments in which the dam was injected *i.v*., metabolism of the labelled cannabidiol may have contributed to the plateau in ratios in these longer experiments (Fig. 4). However, it is worth noting that one main enzyme responsible for hepatic first-pass metabolism of cannabidiol (CYP3A, Jiang et al. 2011) has been shown to have reduced activity in the fetus when compared to the adult (Ochiai et al. 2016), and therefore, metabolism may be less affected at these younger ages.
4. Mode of administration (*i.p.* and *i.v.* versus oral)*.* In human use, cannabidiol is generally administered orally. However, the dose is more difficult to control because of variations in absorption. For this reason, we have chosen to use parenteral administration (*i.v.* or *i.p.*).

## Conclusions and clinical implications

It is important to be careful about extrapolating results from animal studies to humans. However, in this study, we are dealing with fundamental biological mechanisms. Although there may be differences in detail, it is reasonably likely that the overall pattern of results will be significant for pregnant women and their babies. As indicated in the “Introduction” section, there is a wealth of information which allows brain development to be compared between humans and rodents (Clancy et al. 2001; Workman et al. 2013). Also as indicated in the previous section, the placentas in humans and rodents have similar structures.

The studies described here show that in the late gestational rat, the placenta provides a significant impediment to transfer of cannabidiol from the mother to the fetus. Entry of cannabidiol into the brain and CSF at all ages was also demonstrated. The age-related differential expression of cannabinoid receptors in the brain and choroid plexus suggests that there may be different effects of cannabidiol on the developing compared to the adult brain, including effects on behaviour in the offspring of mothers that have taken this drug during pregnancy.

### Supplementary Information

Below is the link to the electronic supplementary material.

Supplementary file1 (PDF 436 kb)

Supplementary file2 (TIFF 883 kb)

Supplementary file3 (TIFF 15354 kb)

## Data Availability

Reasonable requests for data are available from the corresponding author.

## Abbreviations

BCRP: Breast cancer resistance protein
CBD: Cannabidiol
CB1: Cannabidiol type 1 receptor
CB2: Cannabidiol type 2 receptor
CSF: Cerebrospinal fluid
E: Embryonic
P: Postnatal
DPM: Disintegrations per minute
THC: Tetrahydrocannabinol
