# Supplementary material for: Entry of cannabidiol into the fetal, postnatal and adult rat brain
Source: Cell Tissue Res. 2024 Feb 17;396(2):177–95. doi: 10.1007/s00441-024-03867-w (PMC11055756; doi:10.1007/s00441-024-03867-w)
Supplement: Supplementary file 1 — Supplementary file1 (PDF 436 kb) [file 441_2024_3867_MOESM1_ESM.pdf]

Supplementary Information (SI)

Supplementary Figure Legends

Supplementary Fig. 1 Entry of cannabidiol into fetal brain and CSF. 1a, cannabidiol derived via placental transfer from *i.v.* injected dam; 1b, 1c and 1d, cannabidiol injected *i.p.* into individual animals. For all graphs, each point represents a single fetus or pup, bars are Mean ±SD. For all ages data from Fig. 5 subdivided by sex. 1a E19 pregnant rat injected with cannabidiol *i.v.*, 10 mg/kg, n = 10 female, n = 8 male. 1a E19 fetuses injected with cannabidiol *i.p.*, 10 mg/kg, n = 8 female, n = 3 male. 1c P4 day pups injected with cannabidiol *i.p.*, 10 mg/kg, n = 5 for each sex. 1d P12 day pups injected with cannabidiol *i.p.*, 10 mg/kg, n = 3 female, n = 2 male. Note no significant difference between values for male and female for either brain or CSF at any age.

Supplementary Fig. 2 Coomassie stain of PAGE separated plasma at different ages. Note E19 plasma sample was pooled from multiple animals. From left to right samples are: adult (non-pregnant) female, P 12, P4 and embryonic day E19. Molecular standards on the right.

Supplementary Table 1 Dose response of entry of *i.p.* cannabidiol into brain and CSF.

	Cannabidiol Dose (mg/kg)			
	0.1	1	10	50
Cortex	41.4 (35, 48)	38.6 (43, 34)	44.1±4.0 n = 5	58.0 (51, 64)
Brainstem	42.9 (40, 46)	36.6 (52, 22)	49.4±4.6 n = 5	61.5 (56, 67)
CSF	12.1 (18, 6)	20.8 (34, 7)	15.2±2.0 n = 5	38.2 (41, 35)

P12 day rat pup cortex, brainstem and CSF. Values are cortex or brainstem/plasma or CSF/plasma ratio %. Average of n = 2 individual animals at each dose. Individual values in brackets except at 30 min where mean +/- SD, n = 5. Data illustrated in Fig. 1.

Supplementary Table 2 Time course of entry of *i.p.* cannabidiol into brain and CSF.

	Time (min)			
	15	30	60	120
Cortex	31.1 (35, 28)	44.1±4.0 n = 5	50.7 (53, 48)	56.8 (64, 49)
Brainstem	34.4 (36, 32)	49.4±4.6 n = 5	58.2 (62, 54)	62.9 (70, 56)
CSF	8.6 (8, 9)	15.2±2.0 n = 5	31.0 (16, 46)	13.3 (14, 13)

P12 day rat pup 10 mg/kg, brain and CSF. Values are cortex or brainstem/plasma or CSF/plasma ratio %. Average of n = 2 individual animals at each time point. Individual values in brackets except at 30 min where mean +/- SD, n = 5. Data illustrated in Fig. 2.

Supplementary Table 3 E19 Fetus Cortex or brainstem/plasma or CSF/plasma ratio %.

Dam 1

Time (min)	Cortex	Brainstem	CSF
30	73	82	22
36	107	108	29
47	89	96	25
62	117	135	37
70	117	127	35
80	134	140	47
91	102	115	25

Dam 2

Time (min)	Cortex	Brainstem	CSF
30	88	89	20
36	106	106	24
46	85	83	30
62	114	128	40
72	104	97	36

Dam 3

Time (min)	Cortex	Brainstem	CSF
41	117	109	42
56	100	135	21
67	102	130	51
78	117	133	19
90	116	117	35
100	107	101	28

Cannabidiol (10 mg/kg) administered *i.v.* to dams from 30 to 100 min.

Data illustrated in Fig. 5.

Supplementary Table 4 Cannabidiol protein binding to separated plasma protein.

Cut	E19	P4			P12				Adult (non-pregnant) female		
0.5	2.77	9.82	10.67	11.90	15.07	14.65	12.96	19.38	29.61	17.04	13.90
1	2.36	3.12	6.20	10.90	11.59	13.83	8.31	22.66	4.41	6.49	23.18
1.5	6.14	7.61	8.00	6.70	9.76	9.52	12.45	8.19	17.93	7.96	16.39
2	3.21	5.70	13.21	8.62	7.54	9.96	14.53	10.96	16.59	12.73	11.08
2.5	3.30	6.08	18.87	10.98	13.01	16.72	19.33	15.06	12.98	16.84	14.62
3	7.90	11.50	17.23	7.76	16.85	14.25	16.71	13.31	16.78	18.48	11.10
3.5	6.39	8.77	13.68	5.14	11.10	7.17	15.17	6.65	14.56	15.24	5.97
4	2.70	4.97	15.73	5.13	4.88	5.49	14.17	5.22	4.42	14.71	5.71
4.5	2.24	3.04	13.94	4.39	4.02	4.60	12.81	4.75	3.89	13.87	4.33
5	2.05	2.57	11.44	4.05	3.16	3.68	11.20	3.99	3.67	10.03	4.37
5.5	1.69	2.20	10.80	3.26	2.95	2.98	10.58	3.89	2.75	8.11	3.78
6	1.64	2.08	11.66	3.62	2.64	2.69	7.48	3.42	2.89	8.37	3.23
6.5	6.60	1.66	9.76	3.70	3.16	2.87	8.67	4.38	2.03	9.01	3.11

Cannabidiol protein binding to separated plasma protein on SDS-PAGE gels at different ages. Binding of cannabidiol is expressed as disintegrations per minute (DPM) divided by the weight of the gel sample (mg). First gel segment (0.5, italicised) was removed from calculation as it represents large molecular weight compounds that were likely to be long chain fatty acids, which may bind cannabidiol. The ratio of albumin binding at each age was calculated using equation:

$$\text{Albumin Binding (\%)} = \frac{\text{Total Albumin Fraction Bound Counts (DPM/mg)}}{\text{Total Plasma Protein Bound Counts (DPM/mg)}} \times 100\%.$$

Supplementary Table 5 Cannabidiol receptors in brain, choroid plexus and placenta.

GeneID	Symbol	Brain				Choroid plexus				Placenta
		E19	P5	Adult (M)	Adult (F)	E19	P5	Adult (M)	Adult (F)	E19
NM_012784	*Cnr1*	492.1	344.4	297.4	247.7	45.2	11.7	1.0	0.5	0.7
NM_020543	*Cnr2*	0.1	0.1	0.1	0.1	0.1	0.1	0.1	0.1	0.1
NM_001164142	*Cnr2*	0.1	0.1	0.1	0.1	0.1	0.1	0.1	0.1	0.1
NM_001164143	*Cnr2*	0.1	0.1	0.1	0.1	0.1	0.1	0.1	0.1	0.1
NM_012556	*Fabp1*	0.1	0.1	0.1	0.1	0.1	0.1	0.1	0.1	0.1
NM_031982	*Trpv1*	0.8	0.6	1.2	1.3	1.3	1.8	2.0	1.4	7.7
NM_001270798	*Trpv2*	0.1	0.1	0.1	0.1	0.1	0.1	0.1	0.1	0.1
NM_001270797	*Trpv2*	0.1	0.1	0.1	0.1	0.1	0.1	0.1	0.1	0.1
NM_017207	*Trpv2*	0.1	0.1	0.1	0.1	0.1	0.1	0.1	0.1	0.1
NM_001025757	*Trpv3*	0.3	0.5	0.5	0.5	0.2	0.1	0.2	0.2	4.6
NM_023970	*Trpv4*	0.2	0.7	0.7	0.9	78.6	228.1	259.0	286.2	0.5
NM_134371	*Trpm8*	0.7	5.2	0.5	0.4	0.5	0.3	0.2	0.2	1.6
NM_207608	*Trpa1*	0.2	0.4	0.3	0.3	0.2	1.4	0.3	0.7	0.1
NM_053294	*Adora2a*	0.1	0.1	0.1	0.1	0.1	0.1	0.1	0.1	0.1
NM_001357942	*Adora2a*	0.1	0.1	0.1	0.1	0.1	0.1	0.1	0.1	0.1
NM_012585	*Htr1a*	0.9	4.4	5.2	6.6	0.1	0.2	0.1	0.1	0.1
NM_001145367	*Pparg*	0.1	0.1	0.1	0.1	0.1	0.1	0.1	0.1	4.4
NM_001145366	*Pparg*	0.1	0.1	0.1	0.1	0.1	0.1	0.1	0.1	0.3
NM_013124	*Pparg*	0.1	0.1	0.1	0.1	0.1	0.1	0.1	0.1	0.1
NM_012547	*Drd2*	7.3	9.0	3.9	4.8	2.5	0.5	0.3	0.3	0.1

Mean normalised counts per million (CPMs) of all cannabidiol receptors and their variants mined from RNA-sequencing datasets from brain and choroid plexus at E19, P5 and adult (male and female) and E19 placenta. n = 4 for all groups. Standard deviation (SD) is listed in Table 13 for transcripts with expression >1 CPM.
