# Supplementary figures and images for: Entry of cannabidiol into the fetal, postnatal and adult rat brain
Source: Cell Tissue Res. 2024 Feb 17;396(2):177–95. doi: 10.1007/s00441-024-03867-w (PMC11055756; doi:10.1007/s00441-024-03867-w)

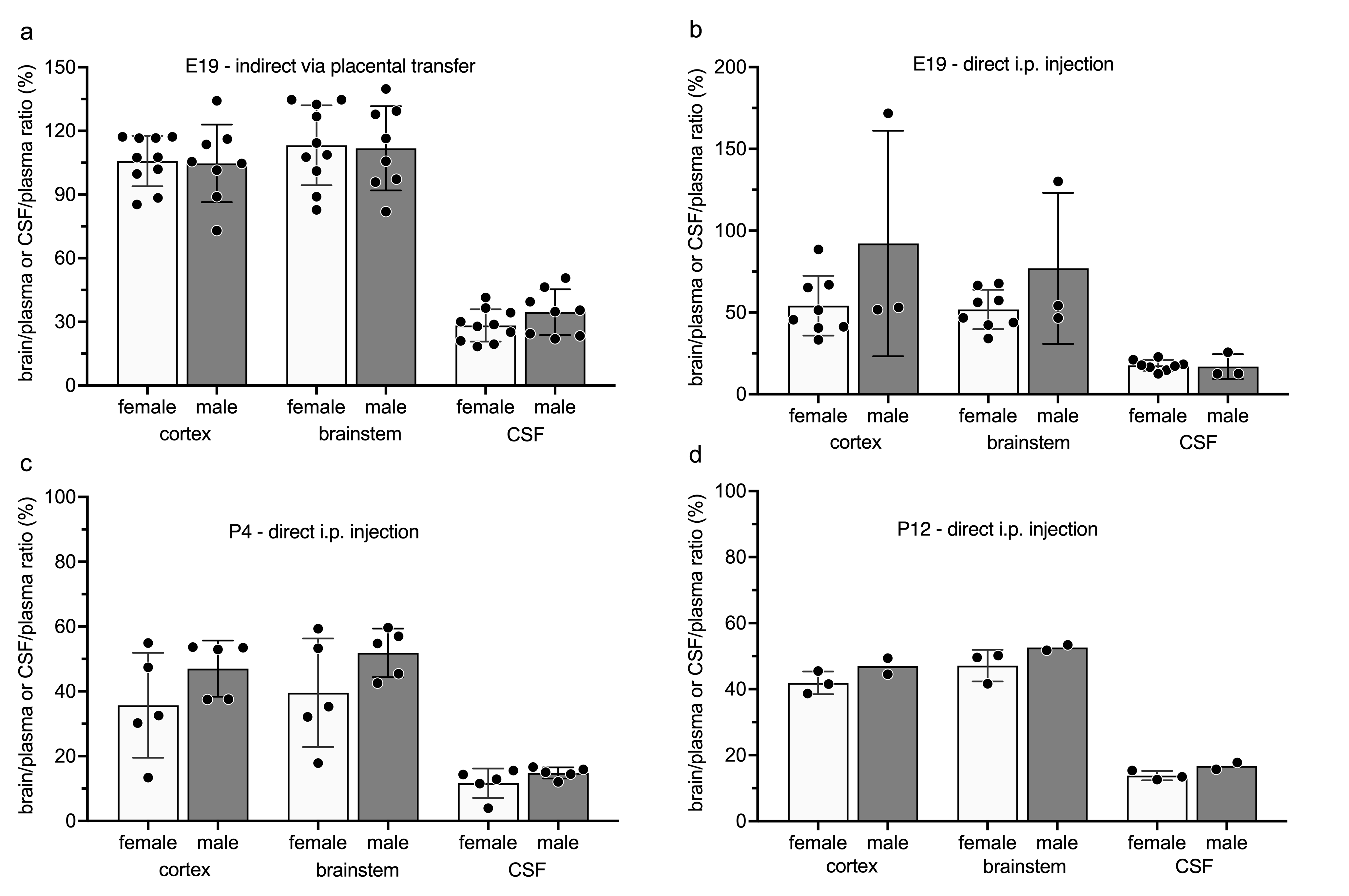

Supplement: Supplementary file 2 — Supplementary file2 (TIFF 883 kb) [file 441_2024_3867_MOESM2_ESM.tiff]

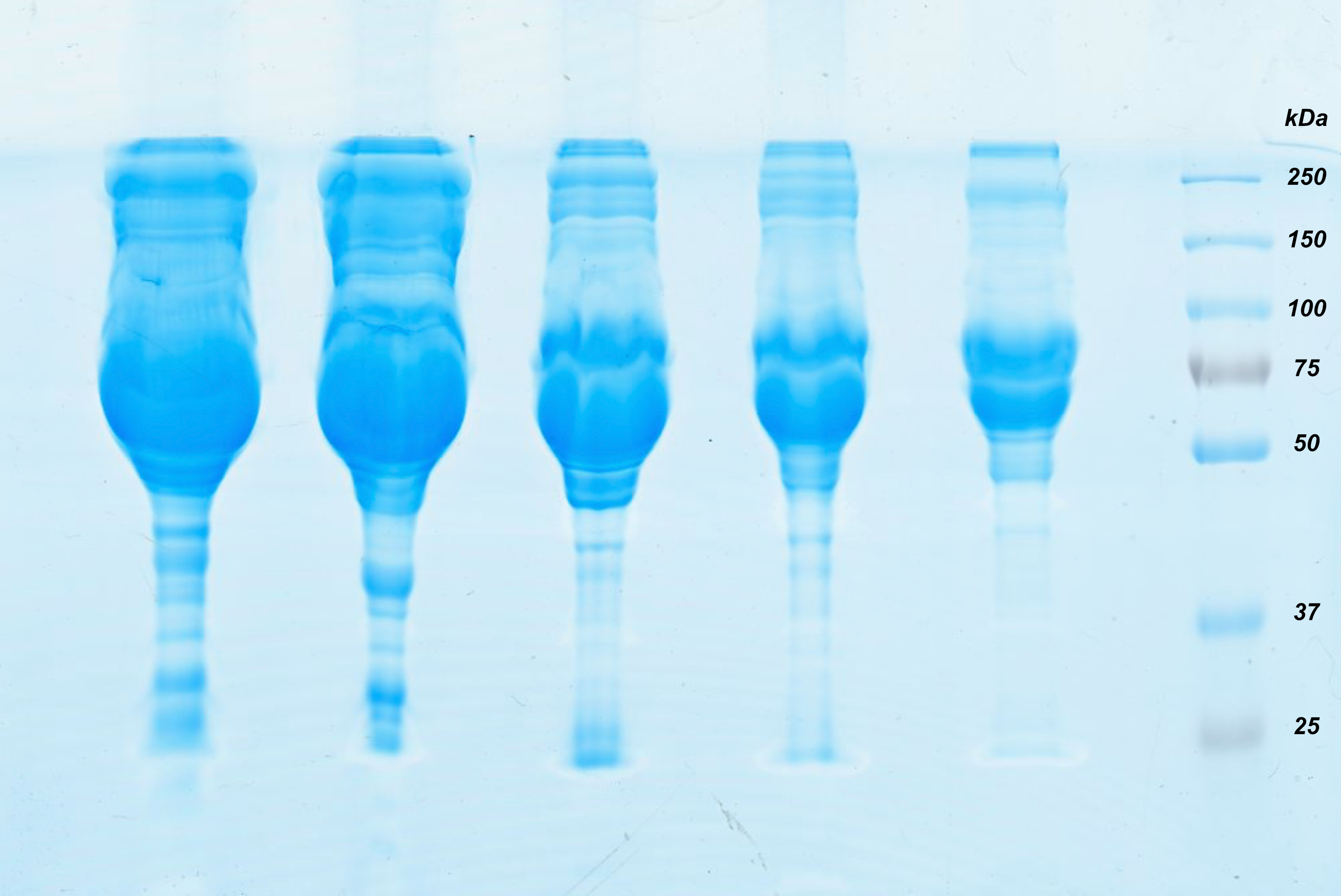

Supplement: Supplementary file 3 — Supplementary file3 (TIFF 15354 kb) [file 441_2024_3867_MOESM3_ESM.tiff]
